# The Best Beef Tea

**Published:** 1872-04

**Authors:** 


### THE BEST BEEF TEA

Is made by boiling a piece of beef slowly, and for several hours, as in making ordinary soup; let it get cold, skim off the grease, boil it again, and then grate the meat into it, which the tea came out of; otherwise there is no nourishment whatever in beef tea alone; some solid food must be mixed with it, then it promotes digestion. Beef has two elements: carbon, or fat; nitrogen, or the thing which gives strength and makes flesh. But if we skim off the fat and take out the meat part, there remains not one particle of nourishment; those who break bread into their soup, or take it with mashed potatoes, or any other solid food, act philosophically; observation has shown them that their soup does them good.

